# Immune-related risk score: An immune-cell-pair-based prognostic model for cutaneous melanoma

**DOI:** 10.3389/fimmu.2023.1112181

**Published:** 2023-02-15

**Authors:** Mingjia Li, Xinrui Long, Wenbo Bu, Guanxiong Zhang, Guangtong Deng, Yuancheng Liu, Juan Su, Kai Huang

**Affiliations:** ^1^ Department of Dermatology, Xiangya Hospital, Central South University, Changsha, China; ^2^ National Engineering Research Center of Personalized Diagnostic and Therapeutic Technology, Changsha, China; ^3^ Hunan Engineering Research Center of Skin Health and Disease, Central South University, Changsha, China; ^4^ Hunan Key Laboratory of Skin Cancer and Psoriasis, Xiangya Hospital, Central South University, Changsha, China; ^5^ National Clinical Research Center for Geriatric Disorders, Xiangya Hospital, Central South University, Changsha, China; ^6^ Department of Dermatology, Peking University First Hospital, Peking University, Beijing, China; ^7^ Department of Dermatological Surgery, Hospital for Skin Diseases, Institute of Dermatology, Chinese Academy of Medical Sciences, Peking Union Medical College, Nanjing, China

**Keywords:** cutaneous melanoma, cell pair, tumor infiltrating immune cell, prognosis model, immunotherapy response

## Abstract

**Background:**

Melanoma is among the most malignant immunologic tumor types and is associated with high mortality. However, a considerable number of melanoma patients cannot benefit from immunotherapy owing to individual differences. This study attempts to build a novel prediction model of melanoma that fully considers individual differences in the tumor microenvironment.

**Methods:**

An immune-related risk score (IRRS) was constructed based on cutaneous melanoma data from The Cancer Genome Atlas (TCGA). Single-sample gene set enrichment analysis (ssGSEA) was used to calculate immune enrichment scores of 28 immune cell signatures. We performed pairwise comparisons to obtain scores for cell pairs based on the difference in the abundance of immune cells within each sample. The resulting cell pair scores, in the form of a matrix of relative values of immune cells, formed the core of the IRRS.

**Results:**

The area under the curve (AUC) for the IRRS was over 0.700, and when the IRRS was combined with clinical information, the AUC reached 0.785, 0.817, and 0.801 for the 1-, 3-, and 5-year survival, respectively. Differentially expressed genes between the two groups were enriched in staphylococcal infection and estrogen metabolism pathway. The low IRRS group showed a better immunotherapeutic response and exhibited more neoantigens, richer T-cell receptor and B-cell receptor diversity, and higher tumor mutation burden.

**Conclusion:**

The IRRS enables a good prediction of prognosis and immunotherapy effect, based on the difference in the relative abundance of different types of infiltrating immune cells, and could provide support for further research in melanoma.

## Introduction

Cutaneous melanoma is a highly malignant tumor derived from melanocytes and is the most invasive and complex of all skin cancers (1). In 2020, the total number of new melanoma cases in the world was 325,000 with 57,000 deaths; these numbers are predicted to increase to 510,000 new cases with 96,000 deaths by 2040 (2). The occurrence of melanoma is caused by interactions between genetic susceptibility and environmental exposure (3), that is, an accumulation of genomic changes, including the mutation burden driven by high-intensity ultraviolet light and prolonged exposure to ultraviolet, which makes melanoma the most immunogenic tumor type with the ability to induce an immune response that can inhibit melanoma growth (4, 5). Immune checkpoint inhibitors, whose main targets are programmed cell death protein 1 (PD1), programmed cell death 1 ligand 1 (PDL1), and cytotoxic T-lymphocyte-associated protein 4 (CTLA-4), have been successfully used in the treatment of melanoma. The total effective rate of immune checkpoint inhibitors in patients with advanced melanoma is 32.9%–58.0% (6). However, only a third of melanoma patients show a durable response to immune checkpoint therapies (7). Biomarkers for the prediction of prognosis and immunotherapy effect in melanoma patients remain elusive. However, previous studies have shown that cytotoxic T lymphocyte (CTL) dysfunction and exhaustion result in lower response and sensitivity to immunotherapy (8). This means that the immune microenvironment is closely related to the effectiveness of immune checkpoint inhibitors.

Tumor-infiltrating immune cells (TIICs), including T cells, B cells, macrophages, and natural killer cells, form an important component of most solid tumors and have an essential role in the host antitumor immune response, which can affect tumor progression *via* antitumor activity or immunosuppression (9, 10). During the process of tumor development, including elimination, balance, and escape, the dual function and plasticity of TIICs lead to complexity and changes in the antitumor response (11, 12). For example, in many tumor types, patients with high levels of CD8^+^ T-cell infiltration tend to have a better prognosis. On the contrary, patients with obvious infiltration of immunosuppressive cells, such as regulatory T cells, tend to have a worse prognosis. Therefore, the quantity and quality of TIICs are key determinants of prognosis (9). The value of TIICs in prognosis prediction and drug resistance analysis has been verified in a variety of tumors, including melanoma (7, 13–15). The American Joint Committee on Cancer (AJCC) guidelines are widely used to evaluate the prognosis of melanoma patients. However, TNM staging mainly describes the invasion and metastasis of tumor tissue at the pathological level, which cannot take into account the composition of tumor-infiltrating cells in the immune microenvironment. Although there are many prognostic models that incorporate immune gene expression, few studies have constructed prognostic models directly based on TIICs. This may be because of the different methods used for determining the specific content of infiltrating cells, which are affected by various measurement factors such that it is difficult to establish a unified standard.

In this study, we develop a prognostic prediction model for melanoma based on TIICs. We adopt the relative value of cell fraction to form a cell pair algorithm. In addition, we present an online nomogram, of which the IRRS is the core, including clinical indicators, to facilitate the use of the IRRS by clinicians.

## Materials and methods

### Study design and data collection

**Figure 1:**
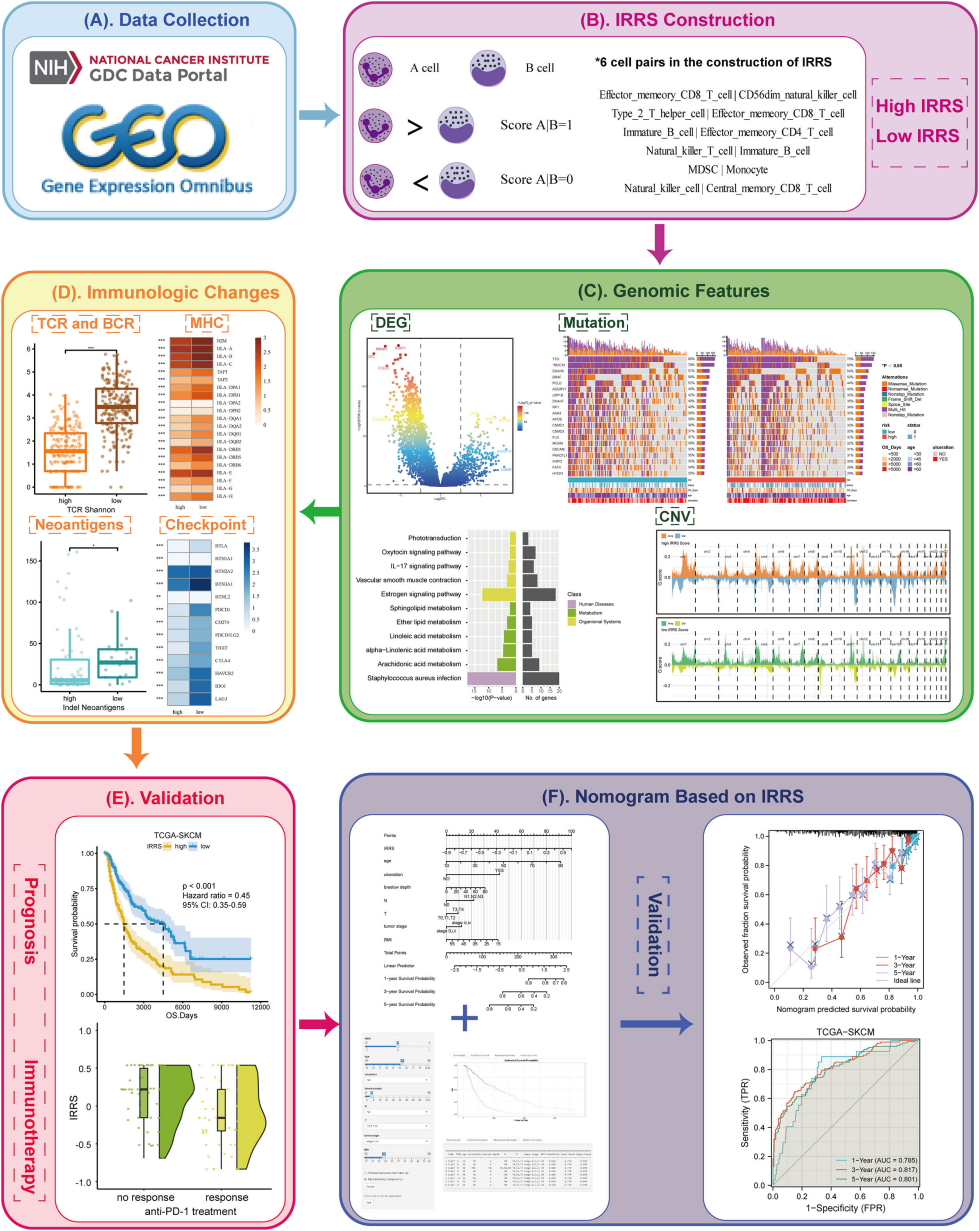
Overview of the workflow. **(A)** The data of this study were from The Cancer Genome Atlas (TCGA) and GEO cohorts. **(B)** The immune-related risk score (IRRS) was constructed by the relative value of cell abundance, that is, cell pairs. **(C)** Discovered the difference in the genomic features between the high and low IRRS through DEG, mutation, and CNV. **(D)** Immunologic changes based on the IRRS was analyzed by the TCR and BCR, MHC, neoantigens, and checkpoints. **(E)** The prognosis and immunotherapy predictive effects were confirmed. **(F)** A nomogram based on the IRRS was constructed and validated.

The integrated research design is presented in **Figure 1**. Transcription profiles and clinical data of cutaneous melanoma patients were obtained from The Cancer Genome Atlas (TCGA; https://portal.gdc.cancer.gov/; TCGA-SKCM cohort). After removing cases with duplication, lack of expression profiles, or lack of survival data, the data of 458 patients were included in the training group for the construction of the IRRS score. The GSE65904, GSE54467, GSE91061, and GSE115821 datasets from the Gene Expression Omnibus (GEO) (https://www.ncbi.nlm.nih.gov/geo/) and a cohort from Liu et al. were used as testing sets for validation (16–20). Missing values in the clinical or pathological data of patients were filled using the missForest package (21–28).

### Establishment and validation of the cell pair algorithm

We carried out single-sample gene set enrichment analysis (ssGSEA) to analyze the expression of corresponding markers of 28 immune cell types (29), thereby obtaining the abundance of these cell types in patient tumor tissues. Then, the cells related to prognosis were screened by univariate Cox (uni-Cox) regression (*P*< 0.05).

The prognosis-related immune cells were termed A cells, and the A cells were paired with all 28 immune cells (termed B cells) in turn to form a set of A|B pairs. If the A-cell abundance exceeded the B-cell abundance for a given cell pair, the value of that pair was recorded as 1; otherwise, it was recorded as 0. This method enables the relative cell abundance to be considered without dependence on the absolute number; this avoids the variation caused by the use of different methods for gene measurement and annotation and differential cell abundance analysis. A matrix containing values of 0 or 1 was constructed, from which cell pairs with 0 or 1 accounting for more than 80% of the total were removed. In the human body, the content of some immune cells is much higher than that of other immune cells, such as neutrophils. The remaining cell pairs were screened by uni-Cox regression analysis (*P*< 0.05) to obtain those correlated with prognosis. We applied the least absolute shrinkage and selection operator (LASSO) Cox regression analysis (glmnet package) to avoid overfitting and obtain the remaining cell pairs. Then, each cell pair was assigned the optimal coefficient by multivariate Cox (multi-Cox), and the IRRS was generated as follows:


$$IRRS=\sum^{}Score\;A|B\times Coef\_A|B$$

The receiver operating characteristic (ROC) curves, the Kaplan–Meier survival curves, the GEO datasets, and the cohort from Liu et al. were used to verify the effectiveness of the IRRS in predicting prognosis and immunotherapy effect.

### Differentially expressed genes and analysis

The differentially expressed genes between the high and low IRRS groups were analyzed using the DESeq2 package, with threshold |log2 fold change (FC)| ≥2 and Benjamini–Hochberg-adjusted *P*-value<0.05 (30). Functional enrichment analysis and clustering of the identified biological processes were conducted using the clusterProfiler R package (31).

The main regulator (MR) is a gene located at the hub of a regulatory network that controls a large number of target genes (termed as its regulon). We used the MR4Cancer tool (http://cis.hku.hk/MR4Cancer) to identify potential MRs that could explain the DEGs between the high and low IRRS groups (32). An MR network diagram was drawn using Cytoscape.

### Genomic features

We used the maftools package to draw the OncoPrint, and the Fischer test was used to evaluate differences in gene mutation frequency between the two groups (33). The somaticInteractions function in the maftools package was used to accurately evaluate the exclusive occurrence and co-occurrence of mutations in pairwise comparisons of the 25 genes with the highest mutation frequency. The DeconstructSigs package was used to analyze the cosmic mutation signature of each patient (34).

Significant deletion or amplification events in the regions of the genome were investigated with GISTIC 2.0, a revised computational program used to identify somatic copy number alterations (35).

### Immunologic changes

T-cell receptor (TCR), B-cell receptor (BCR), and neoantigen data were from the research of Thorsson et al. (36). Tumor immune dysfunction and exclusion (TIDE) score and microsatellite instability (MSI) score were obtained using the official TIDE website (http://tide.dfci.harvard.edu/) (37). The statistical significance of the MSI score was evaluated by Welch’s *t*-test, and other indexes were evaluated by the Wilcoxon rank-sum test.

### Construction and validation of the nomogram model

A nomogram was constructed to predict specific outcomes based on the IRRS and clinical variables using the rms package (38). ROC curves, calibration curves, and decision curve analysis (DCA) curves were drawn to verify the reliability of the nomogram. In addition, the nomogram was compared with the traditional TNM staging system by calculating the integrated discrimination improvement (IDI). Finally, the Dynnom package (cran.r-project.org/web/packages/rms) was used to generate an online version of the nomogram model with an interactive interface for clinical applications.

## Results

### Construction and validation of the IRRS

**Figure 2:**
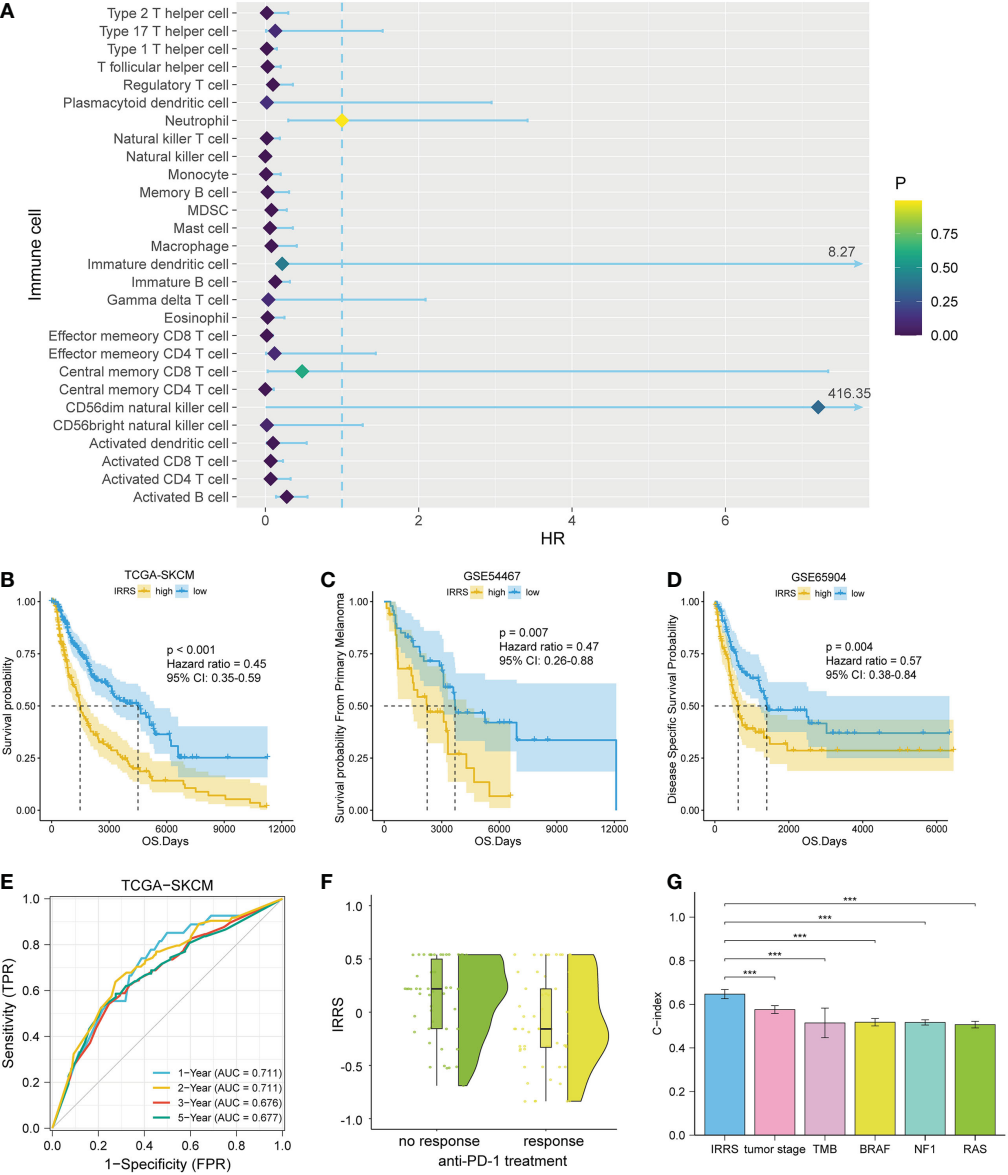
Construction and validation of the IRRS. **(A)** The 19 candidate cells screened based on uni-Cox analysis. **(B–D)** The Kaplan–Meier curves of survival probability for patients in the TCGA-SKCM, GSE54467, and GSE65904 cohorts. **(E)** The ROC curve for patients in the TCGA-SKCM cohort. **(F)** Comparison of immunotherapeutic responses (*P*< 0.01) for patients in the GSE91061, GSE115821, and Liu et al. cohorts. **(G)** Comparison of C-index between the IRRS and tumor stage, TMB, and driver mutations (BRAF, NF1, and RAS) in the TCGA.

A total of 28 immune cell types from 458 melanoma patients (TCGA data) were analyzed. A total of 19 immune cell types related to prognosis were identified by uni-Cox analysis (*P*< 0.05) (**Figure 2A**). After pairing, 532 immune cell pairs were screened and entered into LASSO Cox regression analysis, and 11 immune cell pairs were retained (**Figures S1**, **S2**).

We then carried out multi-Cox regression to generate the best coefficients for the corresponding immune cell pairs; only six immune cell pairs were included in the final model (**Table 1**). Patients with melanoma were classified into high IRRS or low IRRS groups based on the median IRRS score. Compared with patients in the high IRRS group, patients in the low IRRS group had longer overall survival (hazard ratio = 0.45, 95% confidence interval 0.35–0.59, log-rank test *P*< 0.001). To confirm the predictive effect of the IRRS, an ROC curve analysis was performed; the area under the ROC curve (AUC) values were 0.711, 0.711, 0.676, and 0.677 for the 1-, 2-, 3-, and 5-year survival, respectively (**Figures 2B, E** **)**.

**Table 1:** The final immune cell pairs and the corresponding coefficient generated by multi-Cox regression.

Immune cell pairs	Coefficient
Effector_memory_CD8+T_cell|CD56 dim_natural_killer_cell	−0.201895537
Type_2_T_helper_cell_|Effector_memory_CD8_T_cell	0.162775185
Immature_B_cell_|Effector_memory_CD4+T_cell	−0.167620476
Natural_killer_T_cell_|Immature_B_cell	0.376203094
MDSC_|_Monocyte	−0.146018787
Natural_killer_cell|Central_memory_CD8_T_cell	−0.32046159

To further assess the reproducibility and validity of the IRRS, we used external datasets, including GSE65904 and GSE54467, to validate its prognostic value. We also used the median as a group point to plot the Kaplan–Meier curve. Notably, the patients in the high-risk group had shorter overall survival. In addition, in the three anti-PD1 treatment cohorts of GSE91061, GSE115821, and Liu et al., patients with low IRRS exhibited significantly better immunotherapeutic response (*P*< 0.01) (**Figures 2C, D, F** **)**.

### The independent predictive ability of the IRRS

To estimate whether the IRRS was independent of other clinical or pathological factors of melanoma patients, multi-Cox regression was performed, in which covariables included age, gender, body mass index, ulceration, Breslow depth, Clark level, T stage, N stage, M stage, tumor stage, and the IRRS. Multi-Cox analysis showed that the IRRS, age, and ulceration were independent predictive factors for the prognosis of melanoma patients (**Table 2**). The C-index of the IRRS was higher than those of the other independent predictive factors (0.647 for the IRRS *vs*. 0.600 and 0.626 for age and ulceration, respectively). To further confirm the predictive performance of the IRRS, we also compared the C-index values for the IRRS with those for tumor stage, tumor mutation burden (TMB), and driver mutations (BRAF, NF1, and RAS); the results showed that the IRRS had the best predictive effect with respect to prognosis (**Figure 2G**).

**Table 2:** Univariable and multivariable Cox regression analyses of the IRRS and survival in the TCGA cohort.

Characteristics	Total (*N*)	Univariate analysis		Multivariate analysis	
		Hazard ratio (95% CI)	*P*-value	Hazard ratio (95% CI)	*P*-value
IRRS	458	2.718 (2.030–3.639)	**<0.001**	2.899 (2.123–3.959)	**<0.001**
Age	458	1.025 (1.015–1.034)	**<0.001**	1.018 (1.008–1.028)	**<0.001**
Gender	458				
Male	284	Reference			
Female	174	0.878 (0.662–1.164)	0.365		
BMI	458	0.965 (0.931–1.000)	**0.048**	0.985 (0.952–1.020)	0.400
Ulceration	458				
No	214	Reference			
Yes	244	2.523 (1.907–3.338)	**<0.001**	1.970 (1.458–2.662)	**<0.001**
Breslow depth	458	1.026 (1.013–1.040)	**<0.001**	1.006 (0.989–1.024)	0.486
M	458				
M0	435	Reference			
M1	23	1.752 (0.926–3.316)	0.085		
N	458				
N0	277	Reference			
N1, N2, N3	181	1.710 (1.292–2.262)	**<0.001**	1.416 (0.615–3.263)	0.414
T	458				
T0, T1, T2	149	Reference			
T3, T4	309	1.738 (1.301–2.324)	**<0.001**	1.159 (0.843–1.593)	0.363
Tumor stage	458				
Stage 0, I, II	263	Reference			
Stage III, IV	195	1.654 (1.253–2.182)	**<0.001**	1.212 (0.530–2.770)	0.649

The bold values represents P-value < 0.05, that is, the relevant prognostic predictive factors are statistically significant.

**Figure 3:**
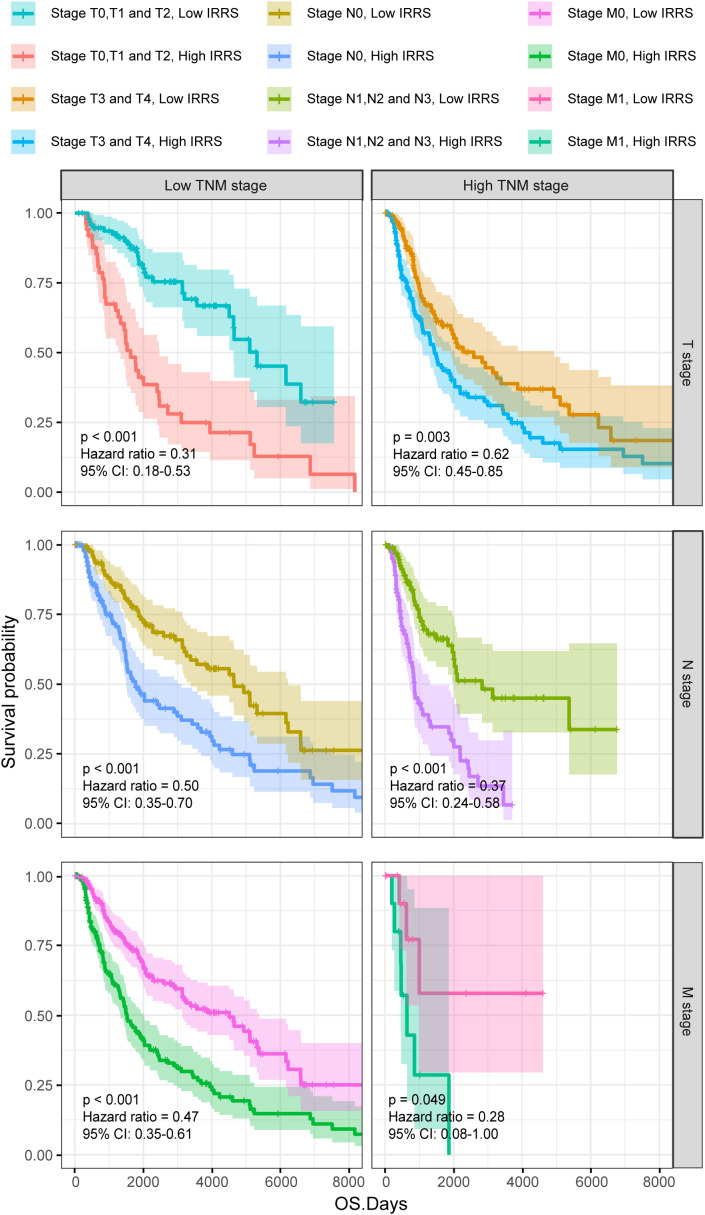
The Kaplan–Meier survival curves according to different TNM stages of patients from the TCGA-SKCM classified into high- and low-risk groups based on the IRRS score.

Furthermore, the high IRRS group had significantly worse overall survival than the low IRRS group, regardless of whether the patients were in the early or late TNM stages (**Figure 3**).

### Enrichment analysis of differentially expressed genes

**Figure 4:**
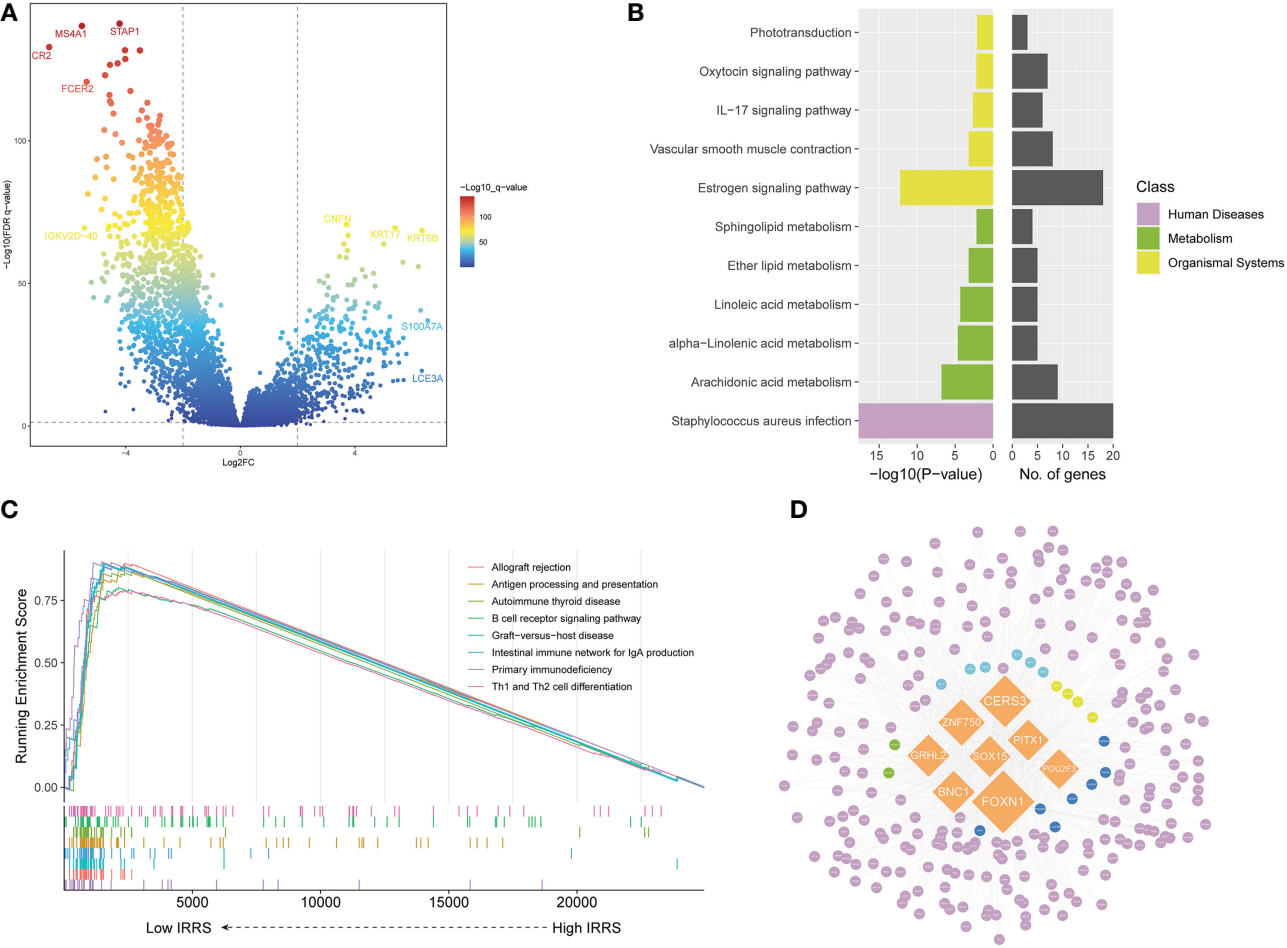
Screening of differentially expressed genes and the master regulator. **(A)** Volcano plot of differentially expressed genes between the low- and high-risk groups in the TCGA cohort. **(B)** KEGG enrichment of differentially expressed genes. **(C)** Gene set enrichment analysis of the high IRRS and low IRRS groups. **(D)** Network of the MRs and DEGs upregulated in the high IRRS group. Orange: eight MRs with the most nodes. Genes related to the KEGG enrichment pathway corresponding to each color: yellow, *Staphylococcus aureus* infection; green, estrogen signaling pathway; blue, both above; dark blue, arachidonic acid metabolism; purple, other DEGs.

Screening identified 422 upregulated genes and 915 downregulated genes in the high-risk group compared with the low-risk group (|log2 FC | > 2, *P*< 0.05) (**Figure 4A**). The Kyoto Encyclopedia of Genes and Genomes (KEGG) pathway analysis of the differentially upregulated genes showed that these genes were mainly enriched in *Staphylococcus aureus* infection and estrogen signaling pathway (**Figure 4B**). In the low IRRS group, GSEA showed significant enrichment, with enrichment scores over 0.7 in 22 pathways, including 12 immune-related pathways. In addition to *S. aureus* infections, some pathways related to viral infection have also been enriched. **Figure 4C** shows the eight immune-related pathways with the highest enrichment scores.

We used MR4Cancer to identify the MRs, which were prioritized based on DEGs through overrepresentation analysis and GSEA. Among them, the eight transcriptional regulators with the most nodes were selected and used to plot a network of the MRs and DEGs from the MRs obtained by the analysis of upregulated DEGs in the high IRRS group (**Figure 4D**). Notably, FOXN1 was found to orchestrate the expression of 236 DEGs.

### Genomic features of the IRRS

**Figure 5:**
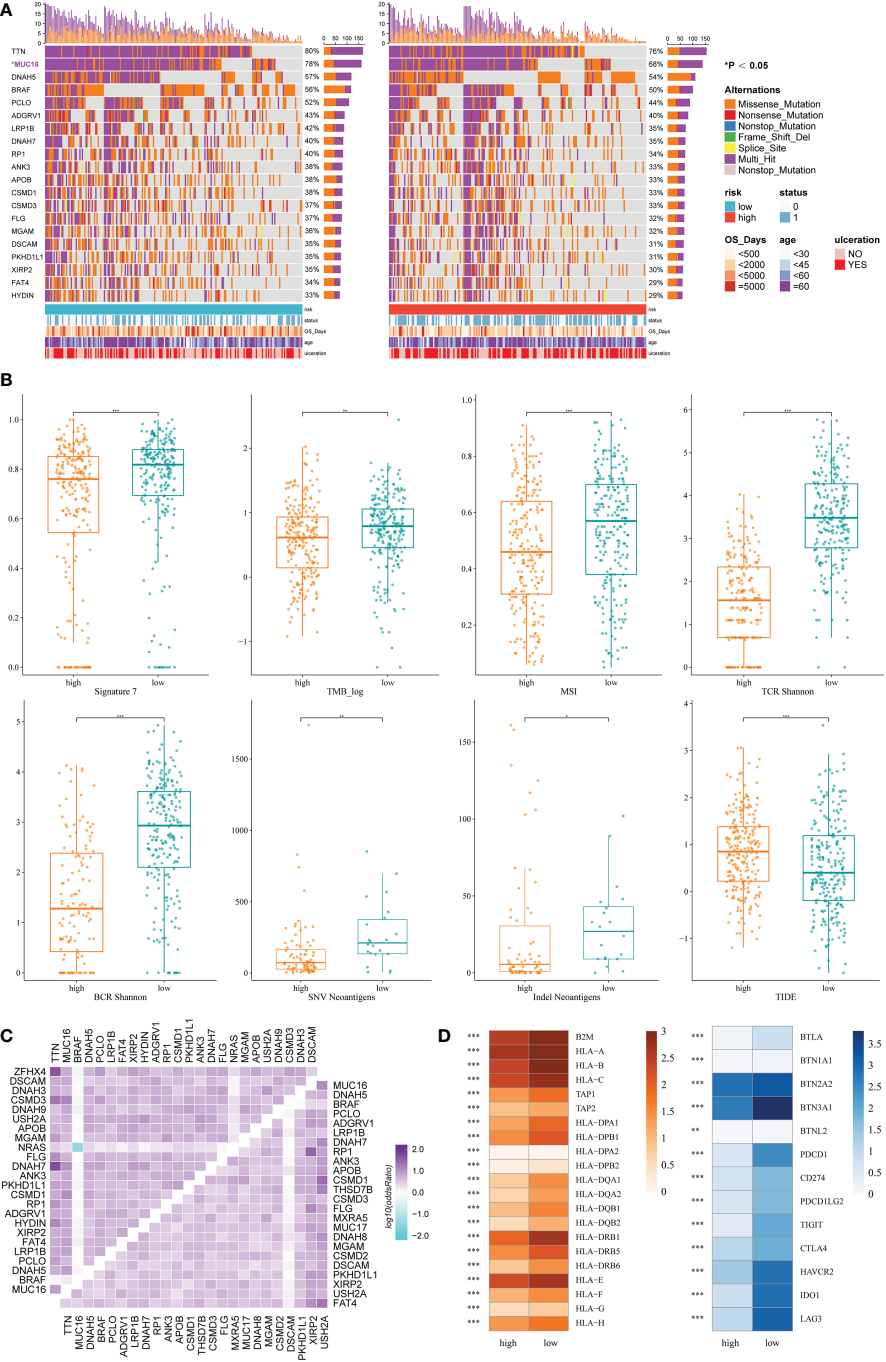
Genomic features and immunologic changes of the high- and low-score groups. **(A)** Mutation of top 20 most frequently altered genes in melanoma patients with high and low IRRS. **(B)** Cosmic mutation signature 7, tumor mutation burden, microsatellite instability, neoantigens, and TIDE score in the high- and low-score groups. **(C)** Heatmap depicting the co-occurrence or exclusivity of the top 25 most mutated genes in the high IRRS group (left upper corner) and the low IRRS group (lower right corner). **(D)** Association between HLA and immune checkpoint molecules and the IRRS. *P<0.05 **P<0.01 ***P<0.001.

Based on the maftools analysis, the top 20 most frequently altered genes were identified (**Figure 5A**). Among these genes, MUC16 was more frequently altered in the low IRRS group, and NRAS–BRAF was the most mutually exclusive pair in the high IRRS group. On the other hand, the most frequently co-occurring gene changes in the low IRRS group were for the pair RP1–MUC16 (**Figure 5C**).

Then, we analyzed the cosmic mutation signatures and found that signature 7 was higher in the low-risk group, which was related to ultraviolet radiation. In addition, the low IRRS group showed higher TMB and MSI (**Figure 5B**).

### The IRRS and immunological mechanism

Immune checkpoint blockade has become a promising strategy for the treatment of many cancers. Therefore, we studied the expression of key immune checkpoint molecules, including PDCD1, CD247, PDCD1LG2, TIGIT, CTLA-4, HAVCR2, IDO1, and LAG3. Cutaneous melanoma patients with low IRRS scores had a higher expression of immune checkpoint molecules, indicating that patients in the low-risk group were more likely to exhibit better immunotherapeutic responses (**Figure 5D**).

We also analyzed adaptive receptors, including TCR and BCR Shannon diversity, and new antigens, including single nucleotide variant (SNV) and indel new antigens. The results showed that the group with low IRRS had higher TCR and BCR diversity and more new antigens (**Figure 5B**). HLA genes control the adaptive immune response by presenting antigens to T cells. The antigen-presenting genes that we analyzed all showed high expression in the low IRRS group (**Figure 5D**).

TIDE uses T-cell dysfunction and exclusion markers to simulate immune escape in tumors with different CTL levels, which can be used to predict the effects of immunotherapy. The high IRRS group had a higher TIDE score, indicating that the patients in the high-score group would have a poorer response to immunotherapy than those in the low-score group (*P*< 0.05).

### Copy number variation

**Figure 6:**
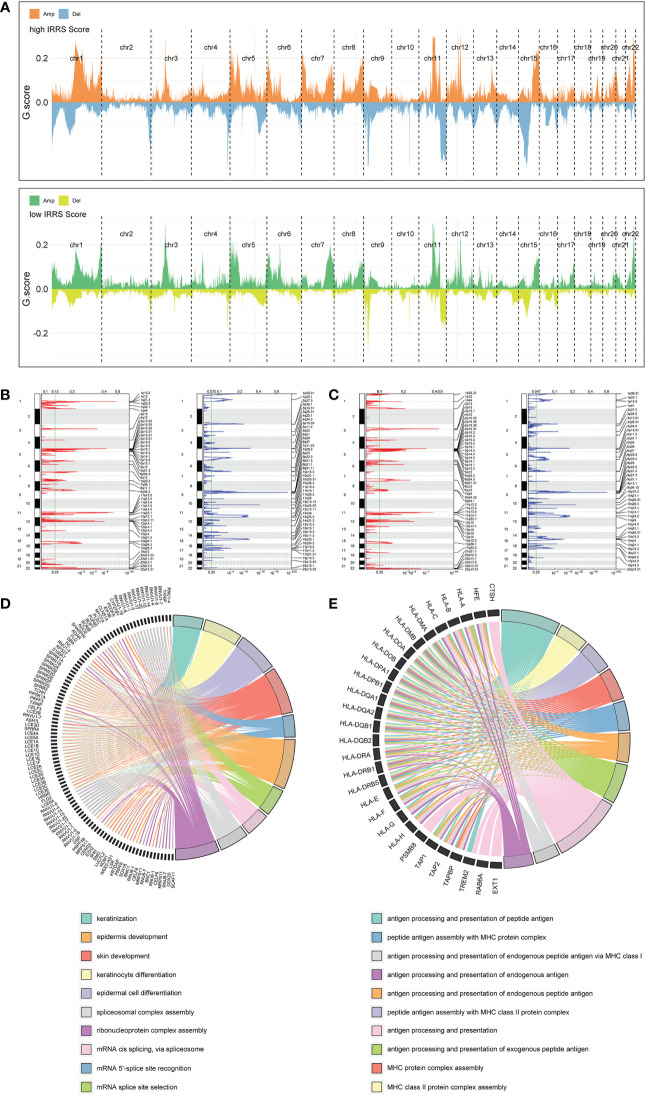
Copy number alterations in the high- and low-score groups. **(A)** Copy number profiles of the high IRRS score (above) and low IRRS score (below) groups. **(B)** Detailed cytobands with focal amplification (red) and deletion (blue) peaks identified in the high IRRS group. **(C)** Detailed cytobands with focal amplification (red) and deletion (blue) peaks identified in the low IRRS group. **(D)** Circular plot of the top 10 biological processes and corresponding enriched genes in the high IRRS. **(E)** Circular plot of the top 10 biological processes and corresponding enriched genes in the low IRRS.

Significant differences in copy number variation were detected between the high IRRS and low IRRS groups (**Figure 6A**). Importantly, focal amplification peaks in some immune-related gene areas were observed in the low IRRS group, such as PDCD1LG2(9p24.1) (**Figures 6B, C**). We annotated specific amplified genes in the high and low IRRS groups through gene ontology biological processes and then clustered the top 10 biological processes. Compared with those in the high IRRS group, the genes amplified in the low IRRS group were more enriched in immune-related processes (**Figures 6D, E** **)**.

### Nomogram based on the IRRS

**Figure 7:**
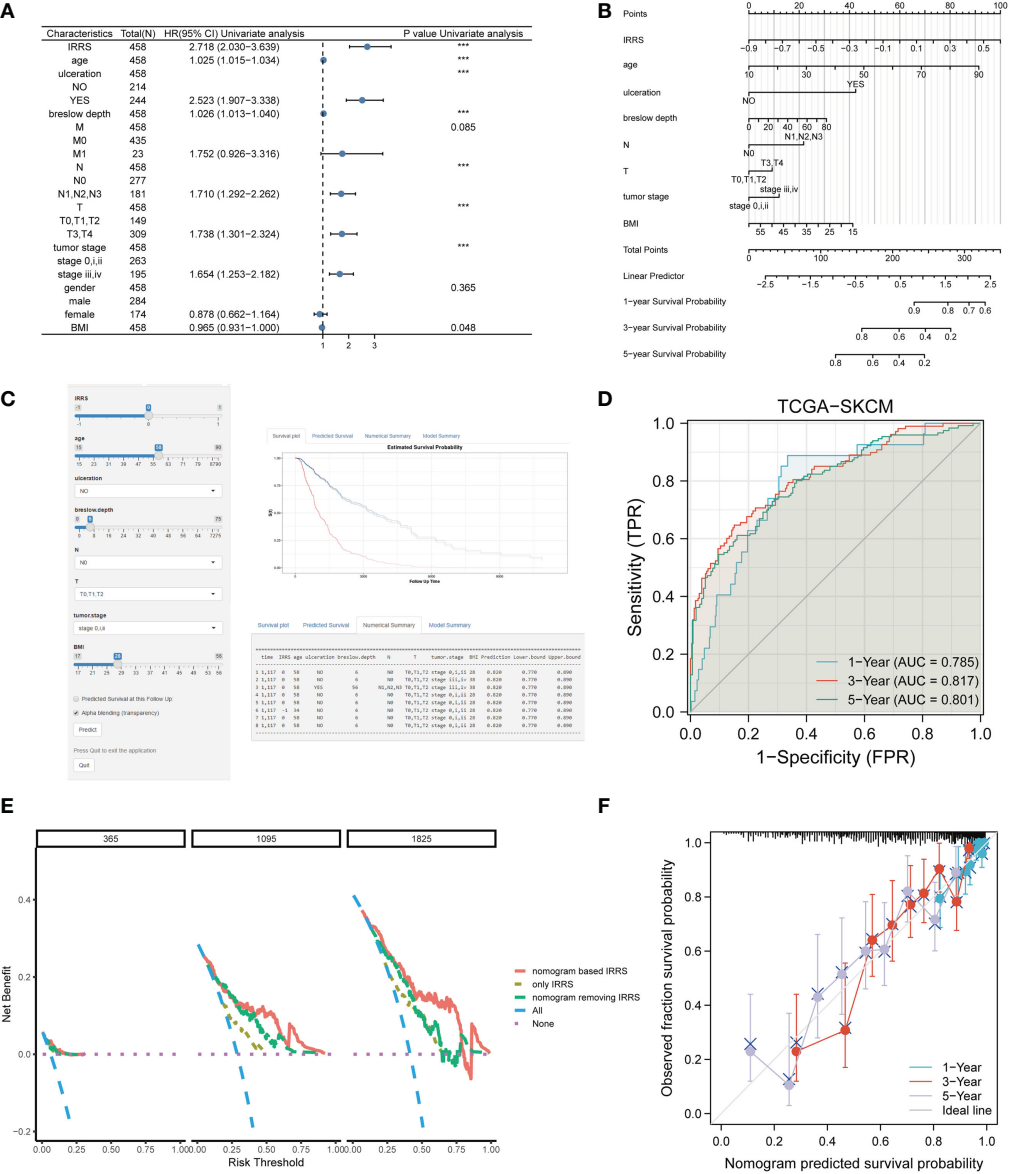
Construction of the nomogram. **(A)** The prognostic clinical factors screened based on uni-Cox regression. **(B)** The nomogram for predicting the survival rate of melanoma patients, including four independent clinical prognostic factors and the IRRS. **(C)** The online version of the nomogram. **(D)** The ROC analysis of the nomogram. **(E)** DCA of the nomogram. **(F)** The calibration curve of the nomogram. ***P<0.001.

Four independent prognostic clinical characteristics associated with overall survival were identified by uni-Cox analysis (*P*< 0.05) and multi-Cox regression (**Figure 7A**). These factors, which comprised age, ulceration, Breslow depth, and N stage, were combined with the IRRS score and used to construct a nomogram to quantitatively estimate the survival rate of patients with cutaneous melanoma (**Figure 7B**).

Although a nomogram can enable visualization of a prognostic model to a great extent, it still requires a ruler to measure continuous variables, which may lead to error because of subjective judgment. Therefore, we developed an online website for clinicians (https://irrsmelanoma.shinyapps.io/IRRSSKCM/), where the predicted survival rate can be easily determined by inputting values of clinical factors (**Figure 7C**).

The calibration results of our nomogram were intuitively consistent with the actual calibration results (**Figure 7F**). The ROC curve analysis indicated that the nomogram had a good predictive effect on prognosis (the AUC values for the 1-, 3-, and 5-year survival were 0.785, 0817, and 0.801, respectively) (**Figure 7D**). According to the DCA curve, the nomogram had the highest overall net benefit within the threshold probability compared with a separate IRRS score or a separate clinical feature (**Figure 7E**). In addition, we compared the nomogram based on the IRRS with traditional AJCC TNM staging; the IDI values for 3, 5, and 10 years were 26.9% (*P*< 0.001), 28.8% (*P*< 0.001), and 32.5% (*P*< 0.001), respectively.

## Discussion

In our study, first, we focused on the influence of TIICs on the prognosis of melanoma patients. Although there have been previous studies exploring the predictive value of immune genes, few prognosis models focusing on the components and content of tumor-infiltrating cells have been established (39–42). Then, we established a cell pair score matrix generated by comparing the abundance of immune cells in pairs, and the IRRS was constructed on the basis of this matrix. Finally, we not only verified the predictive ability of the IRRS but also analyzed its immune and genetic characteristics. An online nomogram integrating the IRRS and clinical information was constructed to avoid errors caused by the measurement process used by clinicians and for further visualization.

The advantages of our model are as follows. First, errors may be introduced into the models by the use of different methods of gene sequencing, continuous updating of annotations, different methods for infiltrating cell analysis, and batch effects; however, the proportion of TIICs exists in a relatively stable range. The adoption of relative values avoids the abovementioned issues. Second, the construction of cell pairs also enables the consideration of interactions between cells and personal immune factors. Therefore, this method improved the predictive ability of the IRRS. In the verification using cohorts from the GEO, the IRRS showed good prediction efficiency with respect to prognosis and immunotherapy response. Through multi-Cox analysis and stratification analysis of the IRRS, we could confirm that the IRRS was independent of other clinical or pathological factors.

In the differential gene expression analysis and KEGG enrichment analysis, the upregulated genes in the high IRRS group were found to mainly affect *S. aureus* infection, estrogen signaling pathway, and pathways related to lipid metabolism (arachidonic acid metabolism, linoleic acid metabolism, etc.). Previous studies have shown that increased colonization of *S. aureus* in squamous cell carcinoma might promote carcinogenesis by inducing chronic skin inflammation (43). Lutchminarian et al. reported a role of pathogenic bacteria in increasing the risk of postoperative complications (44). However, there have been few studies on the direct induction of melanoma carcinogenesis by epidermal microbiota, and whether the change in skin microbiota is the cause or result of melanoma remains to be studied (45). There are gender differences in the incidence of melanoma. The mortality, recurrence, and metastasis rates of melanoma in pregnant women have been found to be higher than those in a non-pregnant control group. Moreover, melanoma-related mortality and sentinel node positivity are higher in women aged 40 to 49 (46). These results suggest that increased estrogen is closely related to the occurrence of melanoma (47). In addition, Conforti et al. confirmed that estrogen could resist the effects of immune checkpoint inhibitors by promoting macrophage polarization (48). A variety of fatty acids are related to the occurrence and development of cancer. An abnormal arachidonic acid metabolic pathway is mainly due to the activation of the COX and LOX pathways, which further affects the occurrence of inflammation and cancer (49). COX-1, COX-2, and LOX are the main drug inhibitor targets of this pathway (50). With the increasing use of immunotherapy, there are excellent prospects for combination treatments involving inhibitors of this pathway acting on specific alkyl receptors (51). Linoleic acid and α-linoleic acid reduce the production of melanin by melanocytes (52). Thus, lipid-related metabolic pathways may represent therapeutic targets in malignant melanoma. In addition, in the high IRRS score group, 236 upregulated DEGs were regulated by FOXN1 as a master regulator. FOXN1 plays an important part in wound healing (53). A possible reason for this upregulation of FOXN1 is that melanoma patients in the high-risk group tend to have worse tumor progression and often develop skin ulceration. Our findings about the MRs may provide new therapeutic targets and potential approaches to treat patients with malignant melanoma.

Antigen presentation ability, tumor immunogenicity, and gene changes can all affect the immune activity of tumors and influence the effectiveness of immunotherapy (54). The high immunogenicity of melanoma makes tumor immunotherapy with checkpoint inhibitors an important treatment option for advanced melanoma patients. The higher TCR, BCR, and HLA diversity in the low IRRS group suggested higher antigen presentation ability in this group. Moreover, the higher levels of SNV or indel neoantigens in the low IRRS were the result of tumor-specific mutations, which determine tumor immunogenicity and increase responsiveness to checkpoint inhibitors (54, 55). In many solid tumors, MSI-H and high TMB are biomarkers of therapeutic benefit (56–58). The low IRRS group had a higher median value for both of these indicators, demonstrating a higher frequency of gene mutation, especially in genes related to ultraviolet exposure (mutation signature 7), which is related to increased sensitivity to checkpoint inhibitor drugs (59, 60). We also analyzed several important immune checkpoints that are related to tumor cell apoptosis (61), T-cell co-inhibition signal, lymphocyte activation (62), and T-cell immunoglobulin mucin (63). The expression levels of immune checkpoints in the high IRRS group were significantly lower than those in the low IRRS group, indicating that the low IRRS group may show a better response to immunotherapy.

In addition, the high IRRS group showed mutual exclusion of NRAS and BRAF. Previous studies have suggested a low incidence of NRAS–BRAF combined mutation, especially in soft tissue malignant melanoma (64). Kumar et al. reported exclusivity between BRAF and NRAS mutations in melanoma, and SPRY4 was a potential mediator of this synthetic response to dual oncogene inhibition (65). Petti et al. showed that the forced expression of NRAS in a single BRAF melanoma line led to growth arrest, that is, when the two mutations coexisted, the viability of cancer cells was impaired (66). On the one hand, this is consistent with our results in the high IRRS score group; that is, there was a higher degree of NRAS–BRAF mutual exclusion in the group with a poor prognosis. On the other hand, the coexistence of double mutations indicates a potential new approach to the treatment of melanoma.

In conclusion, we have introduced the use of relative values, established the IRRS as a prognostic indicator for melanoma, and provided insight into the role of TIICs in the occurrence and development of melanoma and the effects of immunotherapy.

## Conclusion

The IRRS shows a good ability to predict prognosis and immunotherapy effect in melanoma, based on differences in the relative abundance of different types of TIICs, and could provide support for further research in melanoma.

## Data availability statement

The original contributions presented in the study are included in the article/**Supplementary Material**. Further inquiries can be directed to the corresponding author.

## Author contributions

Conceptualization: ML and XL. Methodology: ML, XL and GZ. Acquisition of data: ML, XL, WB, and GD. Data analysis: ML, XL and WB. Validation: YL. Revision of the article: JS, WB, KH and GZ. Writing of the original draft: ML and XL. Visualization: YL, GD and KH. All authors contributed to the article and approved the submitted version.
