# Supplementary material for: Immune-related risk score: An immune-cell-pair-based prognostic model for cutaneous melanoma
Source: Front Immunol. 2023 Feb 15;14:1112181. doi: 10.3389/fimmu.2023.1112181 (PMC9975150; doi:10.3389/fimmu.2023.1112181)
Supplement: Supplementary file 1 [file DataSheet_1.docx]

Supplementary Material

**IRRS, an immune-cell-pair-based prognosis model for cutaneous melanoma**

Mingjia Li^1,2,3,4,5,6+^, Xinrui Long^1,2,3,4,5,+^, Wenbo Bu^7,+^, Guanxiong Zhang^1,2,3,4,5^, Guangtong Deng^1,2,3,4,5^, Yuancheng Liu^1,2,3,4,5^, Juan Su^1,2,3,4,5^, Kai Huang^1,2,3,4,5,*^

*** Correspondence:**Kai Huang Email: kaiserhuang@csu.edu.cn Telephone: 13080564083

^+^These authors contributed equally to this work

# Supplementary Figures


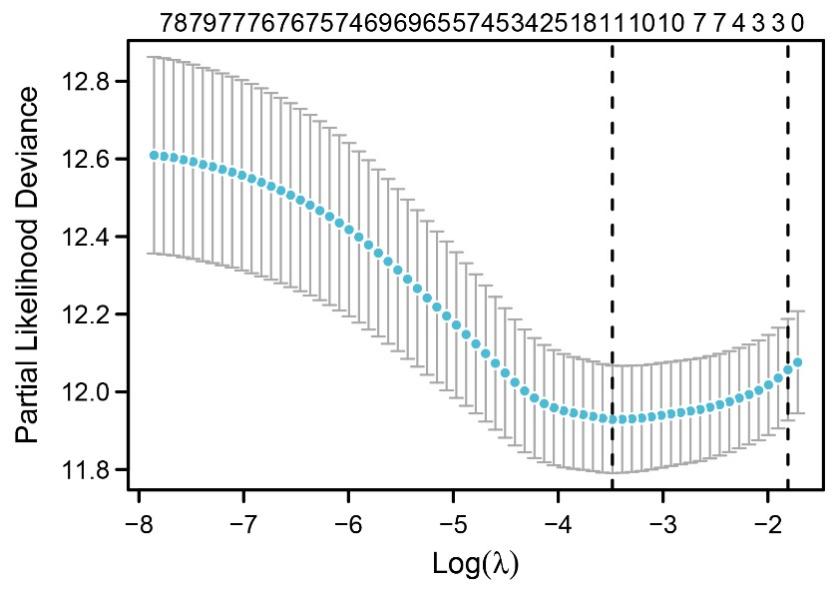


**Supplementary Figure 1.** Tenfold cross-validation for selection of tuning parameters in the LASSO regression. Two dotted vertical lines are drawn at the optimal values according to the minimum criterion (right) and the 1 − SE criterion (left).


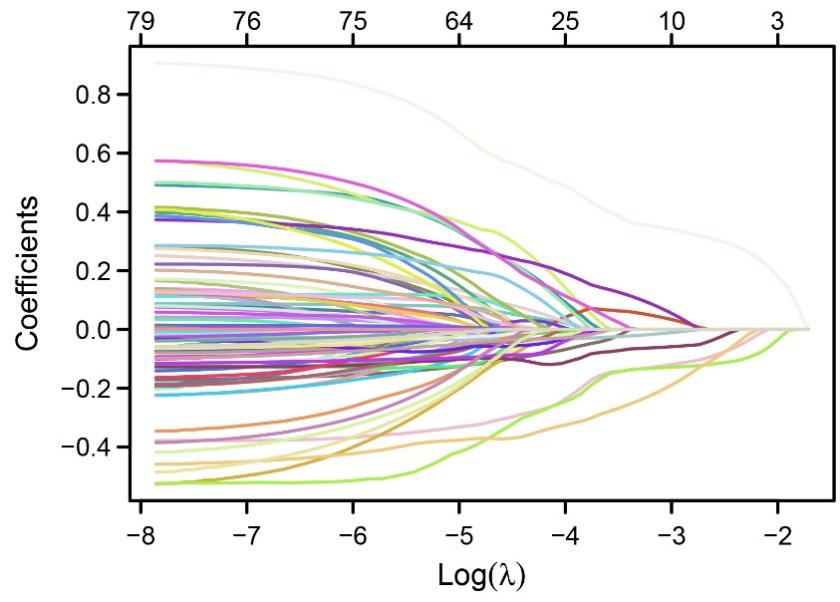


**Supplementary Figure 2.** LASSO coefficient profiles of the 11 candidate cell pairs. The dotted vertical line indicates the optimal value, which was identified by tenfold cross-validation. c.The 19 candidate cells screened based on uni-Cox analysis
